# Simvastatin Alleviates Hyperpermeability of Glomerular Endothelial Cells in Early-Stage Diabetic Nephropathy by Inhibition of RhoA/ROCK1

**DOI:** 10.1371/journal.pone.0080009

**Published:** 2013-11-14

**Authors:** Hui Peng, Pengli Luo, Yuanqing Li, Cheng Wang, Xun Liu, Zengchun Ye, Canming Li, Tanqi Lou

**Affiliations:** Division of Nephrology, Department of Internal Medicine, The Third Affiliated Hospital of Sun Yat-sen University, Guangzhou, P. R. China; University of Houston, United States of America

## Abstract

**Background:**

Endothelial dysfunction is an early sign of diabetic cardiovascular disease and may contribute to progressive diabetic nephropathy (DN). There is increasing evidence that dysfunction of the endothelial tight junction is a crucial step in the development of endothelial hyperpermeability, but it is unknown whether this occurs in glomerular endothelial cells (GEnCs) during the progression of DN. We examined tight junction dysfunction of GEnCs during early-stage DN and the potential underlying mechanisms. We also examined the effect of simvastatin (3-Hydroxy-3-methylglutaryl CoA reductase inhibitor) on dysfunction of the tight junctions of cultured GEnCs and in db/db mice with early-stage DN.

**Methods:**

We assessed the expression of occludin and ZO-1, two major components of the tight junction complex, in cultured rat GEnCs treated with high glucose and in 12 week-old db/db mice with early-stage DN. We also investigated activation of RhoA/ROCK1 signaling, GEnC permeability, and renal function of the mice.

**Results:**

High glucose suppresses occludin expression and disrupts occludin/ZO-1 translocation in GEnCs. These changes were associated with increased permeability to albumin and activation of RhoA/ROCK1 signaling. Occludin and ZO-1 dysregulation also occurred in the glomeruli of mice with early-stage DN, and these abnormalities were accompanied by albuminuria and activation of RhoA/ROCK1 in isolated glomeruli. Simvastatin prevented high glucose or hyperglycemia-induced dysregulation of occludin and ZO-1 by inhibition of RhoA/ROCK1 signaling in cultured GEnCs and in db/db mice with early-stage DN.

**Conclusion:**

Our results indicate that activation of RhoA/ROCK1 by high glucose disrupts the expression and translocation of occludin/ZO-1 and that simvastatin alleviates occludin/ZO-1 dysregulation and albuminuria by suppressing RhoA/ROCK1 signaling during early-stage DN. These results suggest a potential therapeutic strategy for preventing the onset of albuminuria in early-stage DN.

## Introduction

Up to 25% of patients with diabetes have associated kidney damage that can be classified as early-stage or advanced-stage DN [1]. Microalbuminuria is a hallmark of early-stage of DN, and usually indicates damage of the glomerular filtration barrier due to ultrastructural changes in podocytes and glomerular endothelial cells, rather than alterations in glomerular pressure or filtration rate alone [2]. Although dysfunction of glomerular endothelial cells (GEnCs) could be a characteristic of early stage DN [3], [4], the contribution of this dysfunction to microalbuminuria remains unclear.

Studies of diabetic rodent models demonstrated that increased vascular permeability and disruption of vascular integrity occur during the pathogenesis of DN [4]. Other evidence suggested that damage of the endothelial tight junction (TJ) might be a crucial mechanism underlying the increased permeability of endothelial cells [5], [6]. The endothelial TJ is a structural barrier with selective paracellular permeability to solutes and larger molecules [7]. The permeability of the TJ is related to the expression of structural membrane proteins, such as occludin and zonula occludens-1 (ZO-1) [8], [9]. Changes in the localization and expression of occludin/ZO-1 can lead to changes in TJ permeability, especially under pathophysiological conditions. For example, hyperglycemia impairs the expression or function of ZO-1/occludin, and this leads to diabetic retinopathy [5], [6]. However, little is known about the modulation of occludin/ZO-1 and the mechanisms underlying these changes in the pathogenesis of DN.

Previous research has indicated a link between the actin cytoskeleton and occludin/ZO-1, in that signaling molecules that regulate contraction of actin filaments are also important for modulation of TJ permeability [10], [11], [12]. Among these molecules, small GTPases, such as RhoA, have been examined because of their ability to regulate cytoskeletal dynamics [13], [14]. For example, it has been proposed that activation of RhoA/ROCK1 signaling leads to hyper-permeability of GEnCs [15]. Inhibition of RhoA/ROCK1 signaling significantly reduced endothelial damage and vascular leakage that is stimulated by high glucose/advanced glycation end products (AGEs) in cultured endothelial cells and in the presence of diabetes mellitus [16], [17], [18]. All of this evidence indicates a role of RhoA/ROCK1 in disruption of the glomerular filtration barrier in DN.

3-Hydroxy-3-methylglutaryl CoA reductase inhibitors (statins) block cholesterol biosynthesis and are commonly used to treat dyslipidemia. Landmark clinical trials indicate that statins reduce cardiac deaths and vascular disorders in patients with diabetes [19], [20]. However, no clinical study has yet demonstrated that statins benefit patients with advanced-stage DN [21], [22], [23]. This motivated us to investigate the effect of statins on disorders of GEnCs and microalbuminuria in models of early-stage DN.

In the present study, we evaluated the effects of systemic administration of simvastatin on the TJ barrier of GEnCs and on albuminuria in db/db mice with early-stage DN. Moreover, we examined the mechanisms by which simvastatin prevents high glucose-induced TJ dysfunction in cultured GEnCs.

## Materials and Methods

### Chemicals and antibodies

Antibodies against occludin and ZO-1 were from Invitrogen (Carlsbad, CA), anti-GAPDH and anti-β-actin were from Proteintech Group (Chicago, IL), anti-ROCK1 was from Cell Signaling Technology (Danvers, MA), and anti-p-MYPT1 (Thr696) and anti-MYPT1 were from Millipore (Bedford, MA). Simvastatin and mevalonate were purchased from Sigma (St. Louis, MO). Protein A/G PLUS-Agarose was purchased from Santa Cruz Biotechnology (Santa Cruz, CA). Y-27632 (inhibitor of rho-associated kinases) and FITC-BSA were purchased from Sigma-Aldrich (Sigma, MO, USA). Dynabeads M-450 tosylactivated (*φ* 4.5 µm), a magnetic particle concentrator (MPC), Lipofectamine RNAiMAX Reagent, and Lipofectamine LTX/PLUS Reagents were from Invitrogen (Carlsbad, CA).

### Cell culture

Rat glomerular endothelial cells (GEnCs) were established and characterized as described previously [13]. Briefly, GEnCs were grown in RPMI1640 media supplemented with 10% fetal bovine serum and 10% Nu-Serum in a humidified incubator at 37°C with 5% CO_2_. High concentrations of glucose (20–35 mmol/L) commonly occur in the diabetic milieu [24], and many investigators have used this concentration of glucose for *in vitro* endothelial cell experiments [25]. Thus, we simulated severe hyperglycemia in our experiments by use of confluent GEnCs that were serum-deprived for 24 h, and then exposed to a normal level of glucose (NG, 5 mmol/L) or a high level of glucose (HG, 30 mmol/L) for 48 h with or without simvastatin or mevalonate pre-treatment. To inhibit endogenous RhoA, we infected GEnCs with an adenovirus bearing mouse domain negative RhoA (Ad-dnRhoA, kindly provided by Professor Farhad R. Danesh, MD Anderson Cancer Center, Houston TX, USA) for 48 h. Ad-β-galactosidase served as a control.

### Mouse model of DN and tissue preparation

#### Ethics Statement

The animal care and use committee of ZhongShan University reviewed and approved all animal studies.

8 week-old db/db mice (a strain with C57BL6 background, Jackson Laboratory, Bar Harbor, ME) were given oral simvastatin (40 mg/kg/day) for 8 weeks. The mice were housed in individual metabolic cages for collection of urine. Blood glucose was measured using the OneTouch UltraVue Blood Glucose Meter (Lifescan, Milpitas, CA) after a 6 h fast. To assess urinary albumin execration (UAE), 24 h of urine was collected and adjusted to same volume using pure water. Samples of BSA standards or urine were mixed with SDS-loading buffers and boiled for 5 min, and separated by 10% SDS-protein gel electrophoresis followed by Coomassie blue staining (Bio-RAD). The BSA bands (∼65 kD) were scanned and quantified using Image-Pro Plus 6.0 software. To prepare kidney tissue, mice were sacrificed and kidneys were perfused with ice-cold PBS (20 mL/min) with an infusion pump to flush out the blood. Kidneys were cut longitudinally and half of the kidney was stored at −80°C until further examination. The rest of tissue was fixed with 10% buffered formalin phosphate (Fisher Scientific, Hanover Park, IL) overnight and then embedded in paraffin. After preparation of sections (3 µm), slides were deparaffinized and subjected to periodic acid-schiff (PAS) or immunoflorescence staining.

### RNA interference

On-Targetplus siRNA targeting ROCK1 and negative control siRNA (scrambled siRNA) were purchased from Dharmacon (Thermo Scientific, Rockford, IL). Silencing was conducted using the Lipofectamine RNAiMAX Reagent according to the manufacturer's instructions. In brief, plated cells at 70% confluence were incubated with siRNA-Lipofectamine complexes at 37°C for 36 h, and then cells were exposed to normal- or high-level glucose for 48 h.

### Immunoblotting

Cells were treated with lysis buffer (0.3% SDS, 150 mmol/L NaCl, 10 mmol/L Tris buffer, pH 7.4) containing proteinase inhibitors and phosphatase inhibitors (Sigma, St. Luis, MO) for 10 min on ice. Then, cells were collected and centrifuged at 14 000 g for 15 min and the supernatant was subjected to western blotting. The immunoreactive bands were visualized by electrochemiluminescence (ECL) (Millipore) and quantified with Quantity One software (Bio-Rad). For animal studies, the kidney cortexes were washed in cold PBS, lysed in buffer, and prepared for western blotting as described above.

### Transendothelial electrical resistance

The electrical resistance of the confluent GEnCs monolayer was measured with the Millicell-Electrical Resistance System. GEnCs were grown on Corning Transwell filters (Corning, NY). For measurements, the apical and basolateral sides of endothelial cells were bathed in Hank's balanced salt solution (HBSS). Electrical resistance was recorded from probes inserted into the buffer until similar values were obtained on three consecutive measurements. The measured potential difference between the upper and lower wells was used to calculate electrical resistance (Ω/cm^2^). Transendothelial electrical resistance (TEER) values were calculated by subtracting the inherent resistance of the filter and the bathing solution [26].

### GEnCs permeability assay

The rate of BSA flow across a confluent endothelial monolayer was measured as described previously with modifications [27]. Briefly, GEnCs were cultured on a membrane inserted in the transwell (0.4 µm pore size; Corning Inc.). After different experimental treatments, FITC-conjugated BSA (FITC-BSA, 2 mg/mL) was added to the basal-chamber. The fluorescence intensity of the medium in the up-chamber was measured 1 h later using a SpectraMax M5 microplate reader (Molecular Devices, Sunnyvale, CA) with excitation at 492 nm and emission at 530 nm. GEnC permeability was determined by the fluorescence intensity of FITC-BSA passing into the upper-chamber. The results are presented as fold-change after correction with a blank control.

### RhoA activation assay

RhoA activity in GEnC cultures and isolated glomeruli were measured with a RhoA assay kit (Millipore, Bedford, MA) according to the manufacturer's instructions. Briefly, GEnCs were cultured in 75 cm^2^ flasks. After experimental treatment, cells were collected and rinsed with ice-cold PBS, and 500 µL of lysis buffer was added to each dish. Cells were scraped and cell lysates were centrifuged for 5 min at 14 000 g. Supernatants were incubated with a rhotekin RhoA-binding peptide that was immobilized on agarose. Activated GTP-RhoA bound to the rhotekin-agarose was detected by western blotting with a monoclonal anti-RhoA antibody. For the mice study, fresh kidney cortex tissues were washed in cold PBS, lysed in lysis buffer, and treated as described above. Fifteen percent of total cell or tissue lysate from each sample was used as a loading control for RhoA.

### Quantification of mesangial expansion and glomerular volume

Kidney sections (4 µm) stained with periodic acid–Schiff (PAS) were used for measurement of mesangial area and glomerular size. The mesangial index (mesangial area/glomerular area) was determined from digital photos of glomeruli by use of Image-Pro Plus 6.0 software (Media Cybernetics, Bethesda, MD). Glomerular volume (*G_V_*) was calculated based on glomerular area (*G_A_*) as *G_V_* = c× (*G_A_*)^3/2^, in which c was a constant, as previously reported [28].

### Immunofluorescence staining

After treatment with high-dose glucose with or without simvastatin, GEnCs were fixed in 4% formaldehyde solution for 15 min at room temperature, and permeabilized with 0.2% Triton X-100 in phosphate buffered saline with Tween-20 (PBS-T) for 10 min at room temperature. For kidney cryo-sections, slides were permeabilized for 10 min in cold acetone and rehydrated in Tris-buffered saline (TBS). After incubation with the primary antibody and fluorescence-tagging with a secondary antibody, slides were examined by laser scanning confocal microscopy (Zeiss 510 Metaseries, Carl Zeiss Microimaging, Thornwood, NY).

### Isolation of Glomeruli

The procedure used to isolate mice glomeruli was similar to the method described previously [29]. Briefly, mice were anesthetized and perfused with 8×10^7^ Dyna beads diluted in 30 mL of PBS through the heart. Then the kidneys were minced into 1 mm^3^ pieces, and digested in collagenase A (1 mg/mL) in HBSS at 37°C for 30 minutes. The tissue was gently pressed through a 100 µm cell strainer and then rinsed with 3 mL of HBSS. Glomeruli that contained Dyna beads were isolated with a magnetic particle concentrator and washed three times with HBSS.

### Statistical analysis

All data are presented as means and standard deviations. Differences in hyperpermeability of the control and treatment groups were determined with an independent samples *t*-test. The differences between groups were tested with a one-way ANOVA and Fisher's post hoc least significant difference test. All statistical tests were two-sided and a *p*-value of 0.05 was considered significant. All statistical analyses were performed with SPSS version 15.0 (SPSS Inc., Chicago, IL).

## Results

### Simvastatin restored high glucose-induced dysregulation of tight-junctions in cultured GEnCs

**Figure 1:**
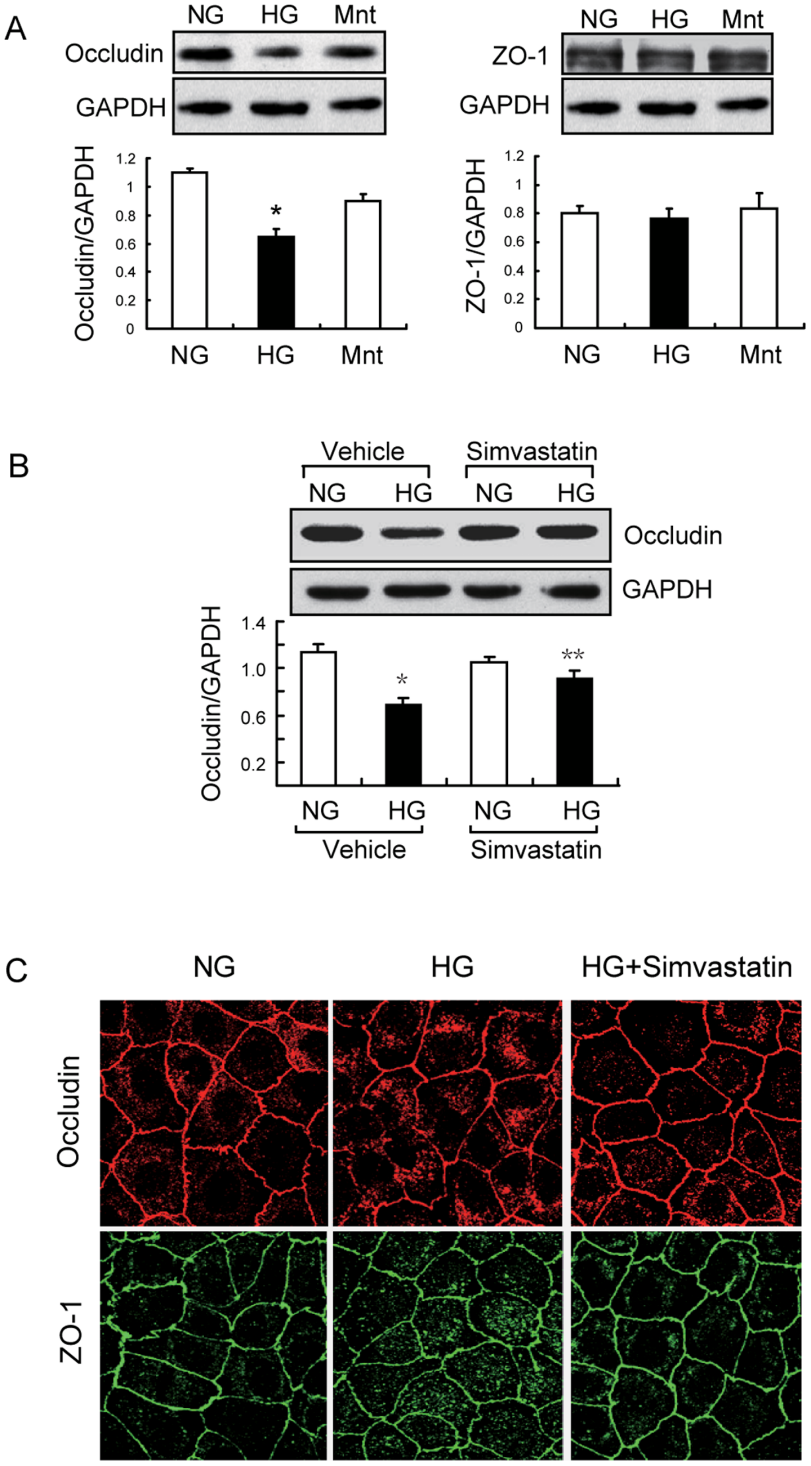
High glucose induces tight-junction dysfunction and simvastatin blunts this response in glomerular endothelial cells. A: Representative immunoblot (top) and GAPDH-normalized band intensity quantification (bottom) for occludin and ZO-1 expression in GEnCs after exposure to High Glucose (HG, 30 mmol/L), normal glucose (NG, 5 mmol/L) and mannitol (Mnt, 30 mmol/L) for 48 h (*n* = 5, *P<0.05 *vs.* NG.). Data are presented as means ± SEMs. B: Representative immunoblot (top) and band intensity quantification (bottom) for occludin expression in response to HG with or without simvastatin (*n* = 5, *P<0.05 versus NG. **P<0.05 *vs*. HG plus vehicle). Glyceraldehyde 3-phosphate dehydrogenase (GAPDH) was an internal loading control. Data are presented as means ± SEMs. C: Consistent with the immunoblot analysis, immunofluorescence microscopy indicated that simvastatin treatment preserves occludin and ZO-1 expression and translocation in the presence of HG.

The endothelial tight junction (TJ) regulates paracellular permeability of endothelial cells, and high glucose (HG) can induce rapid turnover of TJ proteins in retinal endothelial and pigment epithelial cells [30]. Thus, we initially investigated the effect of glucose concentration (NG: 5 mmol/L, HG: 30 mmol/L) on occludin and ZO-1 expression in GEnCs and the effect of simvastatin on expression of these genes under HG conditions. At 48 h after incubation with HG, the expression of occludin was significantly suppressed compared with cells treated with NG (Figure 1A, left panel). However, ZO-1 expression was the same under NG and HG conditions (Figure 1A, right panel). The response we observed was not due to the high osmolarity of HG, because mannitol (25 mmol/L) did not alter occludin/ZO-1 expression.

We then examined the effects of simvastatin on occludin expression. The results indicate that simvastatin significantly blocked HG-induced suppression of occludin, but did not alter the expression of occludin under NG conditions (Figure 1B). Translocation of occludin/ZO-1 is critical for TJ formation and function [31], so we assessed the distribution of occludin and ZO-1 in response to HG by use of immunofluorescence staining. As shown in Figure 1C, occludin expression was continuous along the cellular borders under NG conditions, but had a weak and patchy expression pattern after 48 h under HG conditions. In addition, the fraction of cytoplasmic occludin and ZO-1 were significantly increased, indicating that HG disrupted the translocation of occludin/ZO-1. Notably, simvastatin partially reversed both of these responses to HG (Figure 1C). These results demonstrated that HG disrupts TJ formation in GEnCs and that simvastatin treatment prevents HG-induced TJ dysregulatin by restoring occludin expression and occludin/ZO-1 membrane translocation.

### Simvastatin reversed high glucose–induced hyper-permeability in glomerular endothelial cells (GEnCs)

**Figure 2:**
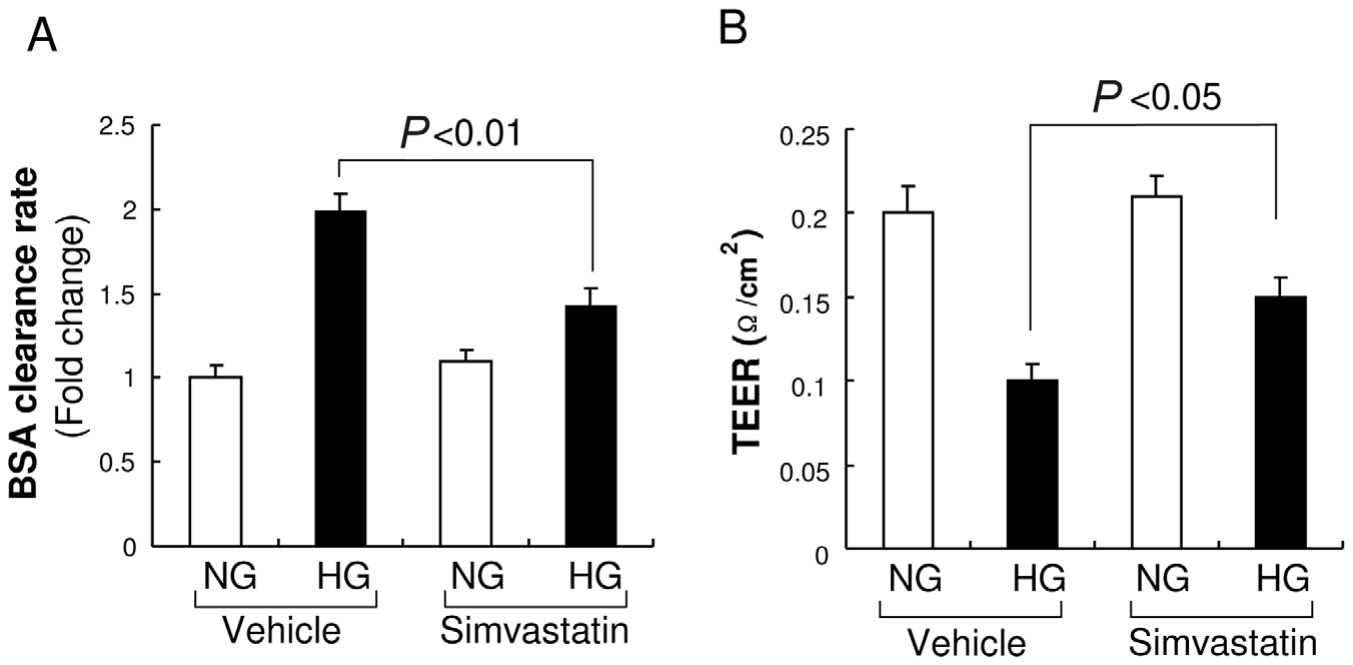
High glucose increases permeability of glomerular endothelial cells (GEnCs) and this effect is inhibited by simvastatin. A: GEnC permeability was determined by measuring the amount of FITC-albumin that crossed the GEnCs monolayer during first hour after FITC-albumin (2 mg/mL) was added. High glucose (HG) increased permeability in GEnCs, as indicated by the rate of BSA transfer across the GEnCs monolayer, and simvastatin reduced this effect (*n* = 7). Data are presented as means ± SEMs. B: GEnC permeability was measured by transendothelial electrical resistance (TEER). HG reduced the TEER of the GEnC monolayer and simvastatin inhibited this response (*n* = 7). Data are presented as means ± SEMs.

HG apparently induces GEnC hyperpermeability *in vitro*, so we examined the effect of simvsatatin on this response in cultured GEnCs. First, we validated our GEnC permeability assay by using a trans-well system and measurement of the rate of albumin movement across the GEnC monolayer after exposure to HG for 48 h. As shown in Figure 2A, HG increased albumin permeability of GEnCs compared with NG. We then examined the effect of simvastatin on the hyper-permeability induced by HG. The results indicated that simvastatin significantly reduced HG-induced hyperpermeability, and that simvastatin alone did not alter the permeability of GenCs when co-incubated with NG. We next confirmed the effect of simvastatin by measuring the transendothelial electrical resistance (TEER), an alternative indicator of permeability. As expected, HG reduced the TEER of the GEnC monolayer and simvastatin markedly reversed this response (Figure 2B). Thus, HG increases the permeability of GEnCs and simvastatin blocks this response in cultured GEnCs.

### RhoA/ROCK1 signaling is involved in regulation of occludin expression and translocation in GEnCs

**Figure 3:**
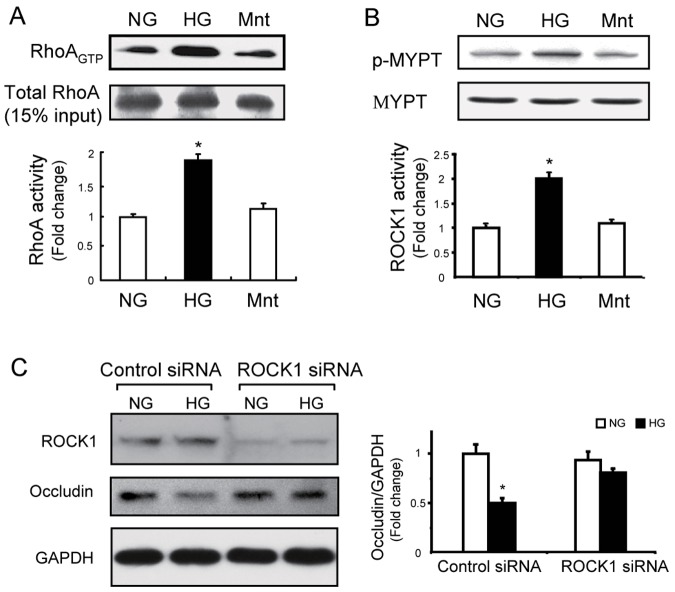
RhoA/ROCK1 signaling mediates occludin expression and translocation in glomerular endothelial cells (GEnCs). A: GEnCs were incubated with high glucose (HG, 30 mmol/L), normal glucose (NG, 5 mmol/L), or mannitol (Mnt, 30 mmol/L) for 48 h. RhoA activity was assessed by the PEK-agarose pull-down assay, as described in the Materials and Methods. A total of 15% of total protein lysate from each sample served as loading controls (*P<0.05 HG *vs*. NG; *n* = 5). B: ROCK1 activity was assessed by measuring the p-MYPT1 (Thr696)/MYPT1 ratio in GEnCS with same treatment as in Figure 3A (*P<0.01 HG *vs*. NG; *n* = 5). C: GEnCs were incubated with ROCK1-siRNA for 36 h to knockdown ROCK1 (upper panel); scrambled siRNA served as the control. The cells were exposed to normal glucose (NG) or high glucose (HG) for 48 h and occludin expression was assessed using immunoblotting, with GAPDH as a loading control (*P<0.05 HG *vs.* others; *n* = 5).

Previous research indicated that small GTPases, especially RhoA and its downstream effector ROCK1, regulate TJ formation [32]. Thus, we examined the effects of HG on RhoA and ROCK1 activation. The results indicated that HG treated cells had significantly increased RhoA binding to GTP relative to NG treated cells (Figure 3A). ROCK1 was also activated because the phosphorylation rate of MYPT1 (a downstream effector of ROCK1) was significantly increased by HG treatment (Figure 3B). These results demonstrated that activation of RhoA/ROCK1 signaling is associated with HG-induced TJ dysfunction in GEnCs. To test whether RhoA/ROCK1 signaling mediates HG-induced occludin loss and impedes translocation, we used siRNA to knockdown ROCK1 in GEnCs. The results indicated that ROCK1 inhibition did not influence the expression of occludin under NG conditions, but in presence of HG, knockdown of ROCK1 resulted in increased occludin expression (Figure 3C). These results indicate that HG induces TJ dysfunction through RhoA/ROCK1 signaling.

### Simvastatin prevents HG-induced ROCK1 activation in cultured GEnCs

**Figure 4:**
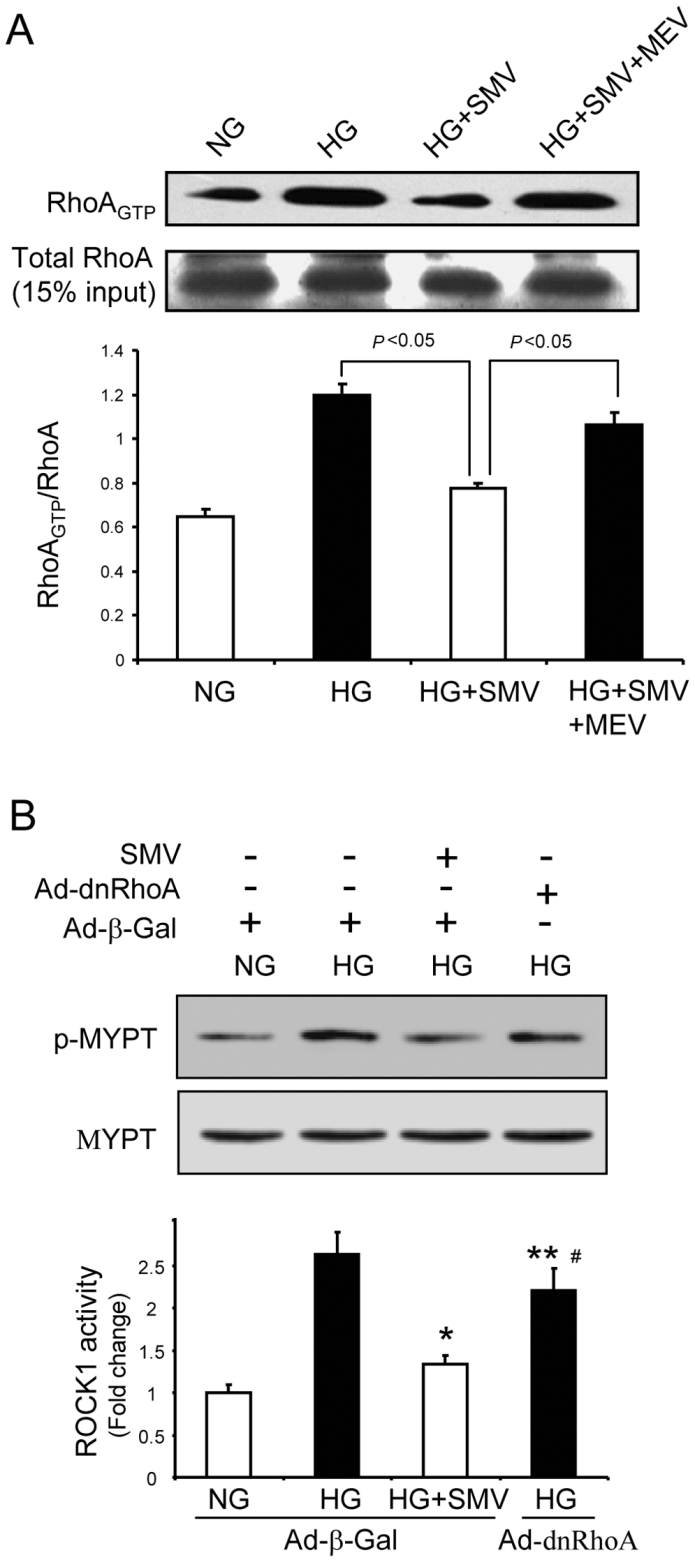
Simvastatin inhibits ROCK1 activation stimulated by high glucose in GEnCs. A: GEnCs exposed to high glucose (HG) were treated with simvastatin (SMV) or simvastatin plus mevalonate (MEV) for 48 h. RhoA activity was examined using the PEK-agarose pull-down assay. Fifteen percent of total protein lysate from each sample served as the loading control Data are presented as means ± SEMs (*n* = 5). B: GEnCs were infected with adenovirus encoding beta-galactosidase (Ad-beta-Gal, served as control) or domain-negative RhoA (Ad-dnRhoA). After 24 h, cells were incubated in NG or HG medium for 48 h. In some experiments, simvastatin (SMV) was added as indicated. The p-MYPT1 (Thr696)/MYPT1 ratio was used to assess ROCK1 activity (*P<0.01 HG+SMV *vs.* HG; ** *P*<0.01 HG+dnRhoA *vs.* HG+SMV; #<0.05 HG+dnRhoA *vs.* HG). Data are presented as means ± SEMs (*n* = 5).

Next, we examined the effect of simvastatin on RhoA/ROCK1 signaling in GEnCs exposed to HG media. Simvastatin partially normalized the HG-induced activation of RhoA, and this response was reversed by addition of mevalonate (MEV), the product of HMG enzyme. This indicates that the protective effect of simvastatin is due to its inhibition of mevalonate biosynthesis (Figure 4A). Interestingly, comparison of the effects of simvastatin and a domain-negative mutant RhoA (dnRhoA) on ROCK1 activation in cultured GEnCs indicated that overexpression of dnRhoA partially blunted HG-induced ROCK1 activation, and this effect was smaller than that of simvastatin (Fig 4B). This result indicated that simvastatin not only suppresses RhoA-dependent ROCK1 activation, but also blocks RhoA-independent ROCK1 activation. Taken together, these *in vitro* studies indicated that simvastatin prevented the HG-induced hyperpermeability of GEnCs and that restoration of TJ function via suppression of RhoA/ROCK1 signaling is at least partially responsible for this effect.

### TJ dysfunction is associated with albuminuria in db/db mice with early-stage DN

**Figure 5:**
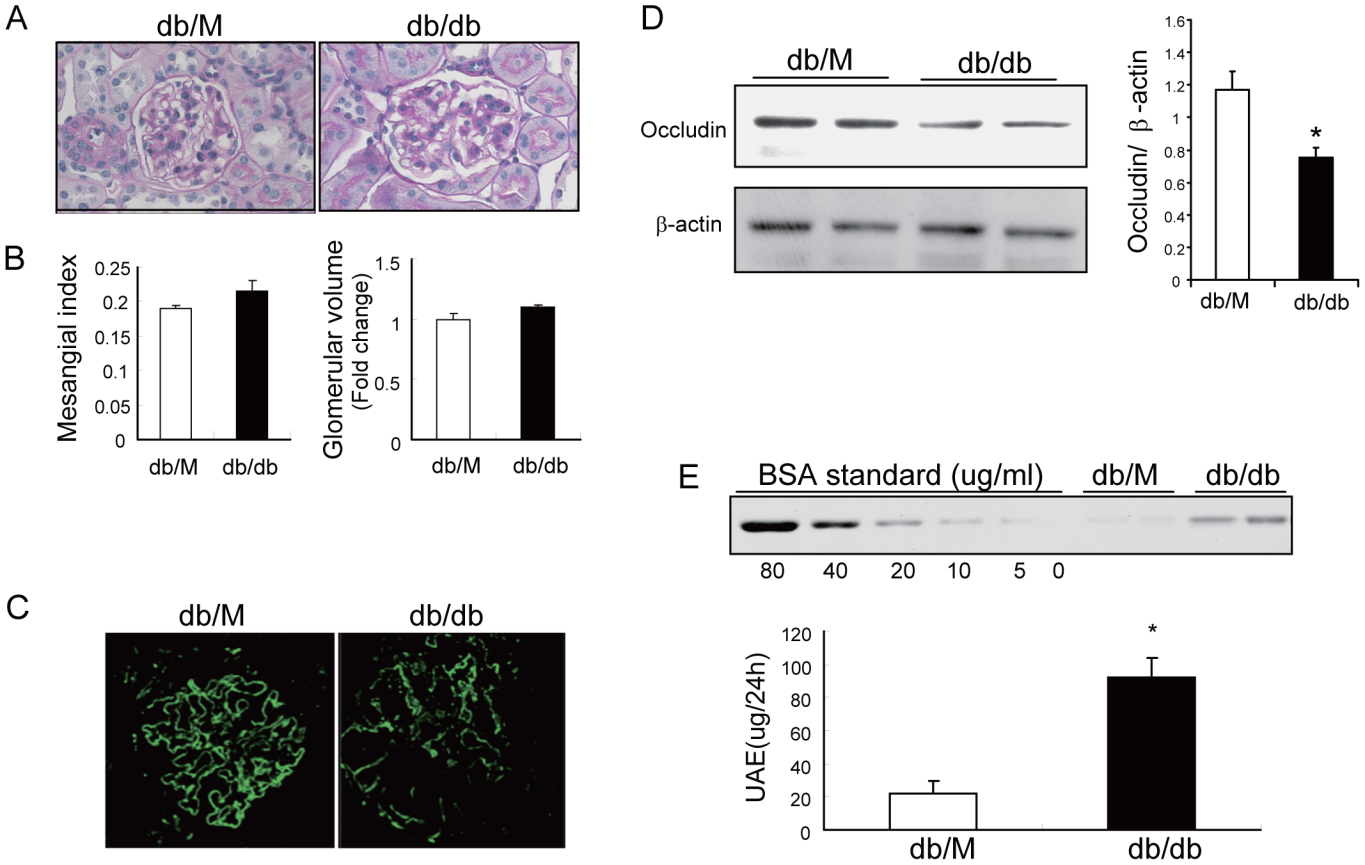
TJ dysfunction and albuminuria occur in db/db mice with early-stage diabetic nephropathy. A: Representative PAS (periodic acid–Schiff) staining of 3 µm kidney sections of 12 week-old db/m (control) and db/db mice at 400× (top), B: mesangial index and relative glomerular volume have no significant differences between db/M and db/db mice. Data are presented as means ± SEMs (*n* = 7). C: Kidney sections (3 µm) of db/M and db/db mice were stained with anti-occludin and examined by confocal microscopy (400×). D: Isolated glomerular lysate (30 µg per sample) was subjected to immunoblotting to assess occludin expression. beta-actin was used as a loading control (*P<0.05 db/db *vs.* db/M). Data are presented as means ± SEMs (*n* = 7). E: 24 h urinary albumin execration (UAE) was determined by PAGE and results are presented as ug/24h (*P<0.01 db/db *vs.* db/M). Data are presented as means ± SEMs (*n* = 7).

We used 12 week-old db/db mice with a C57BL6 background and assessed the expression of occuldin and ZO-1 to determine if TJ dysfunction occurs during early-stage DN. Use of this strain was based on previous studies, which indicated development of albuminuria prior to significant mesangial expansion at 12 weeks of age [33]. The histological analysis indicated no obvious changes in mesangial area or glomerular size of db/db mice compared with db/M (control) mice (Figure 5A and B). Immunofluorescence microscopy revealed that the pattern of occludin was smooth and linear in glomeruli of control mice, however, there was decreased occludin expression in the glomeruli of db/db mice, and the expression had a granulated pattern (Figure 5C). Western blotting analysis confirmed the reduction of occludin in isolated glomeruli of db/db mice (Figure 5D). Associated with the dysregulation of TJ components, 24 h urinary albumin excretion (UAE) was significantly increased in db/db mice (Figure 5E). These results indicated that TJ dysfunction occurs in GEnCs during early-stage DN.

### Simvastatin ameliorates albuminuria in mice with early stage of DN

**Figure 6:**
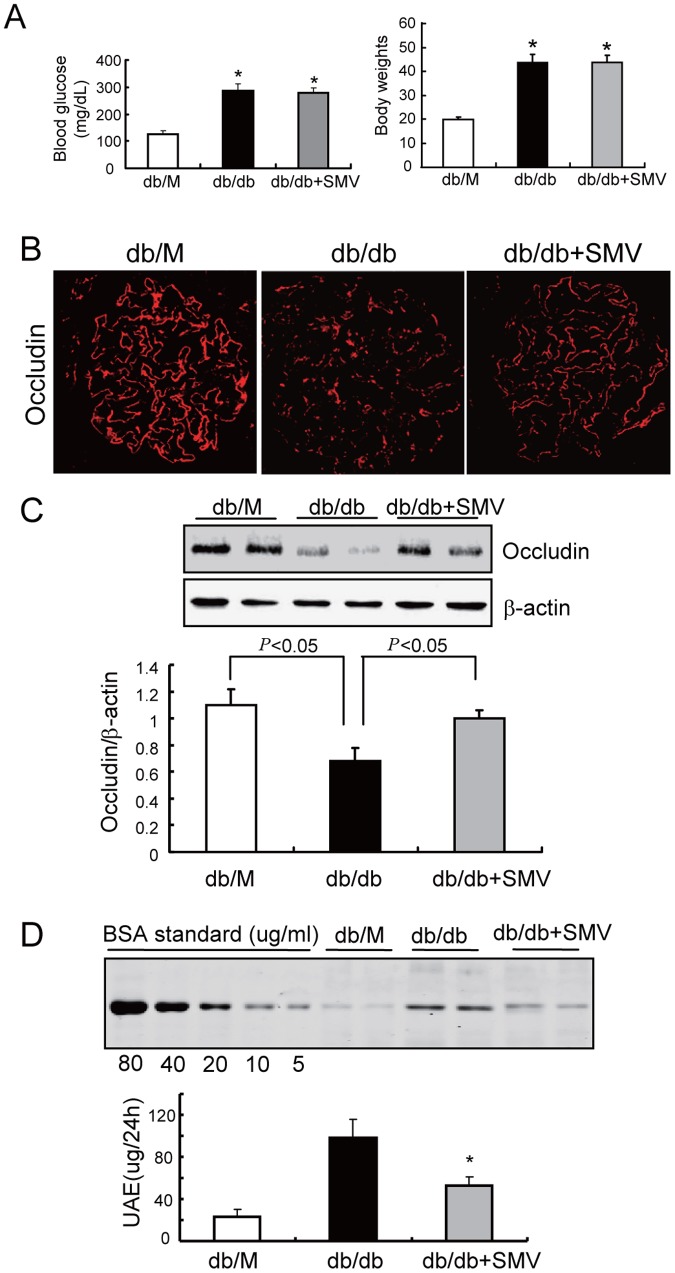
Simvastatin restores occludin expression and ameliorates albuminuria in mice with early-stage DN. A: Twelve week-old db/m and db/db mice were given simvastatin (SMV, 200 mg/kg, ig) for 4 weeks. Before kidney harvest, blood glucose and body weight were recorded (*P<0.01 *vs.* db/m). Data are presented as means ± SEMs (*n* = 12). There were no significant differences between db/db and db/db + simvastatin mice. B: Occludin distribution and expression was assessed in kidney sections by immunofluorescence microscopy. Representative images are shown at 400×. C: Occludin expression was also examined by immunoblotting of isolated glomerular lysate, with beta-actin as a loading control. Data is presented as means ± SEMs (*n* = 7). D: Urine from mice (Figure 6A) was collected for 24 h and urinary albumin excretion (UAE) was determined using PAGE. Results are presented as ug/24 h (*P<0.01 db/db+SMV *vs.* db/db). Data are presented as means ± SEMs (*n* = 12).

We then examined the response of db/db mice to simvastatin treatment. After 8 weeks of treatment, blood glucose and body weight were not significantly different in untreated db/db mice and simvastatin-treated db/db mice (Figure 6A). However, the dysregulation of occludin in the glomeruli was largely reversed by simvastatin (Figure 6B and C), and this protective effect was associated with a significant decrease in albuminuria (Figure 6D). These results indicated that simvastatin prevents occludin dysregulation in GenCs and the onset of albuminuria during early-stage DN.

### Simvastatin prevented ROCK1 activation in glomeruli of mice with early stage of DN

**Figure 7:**
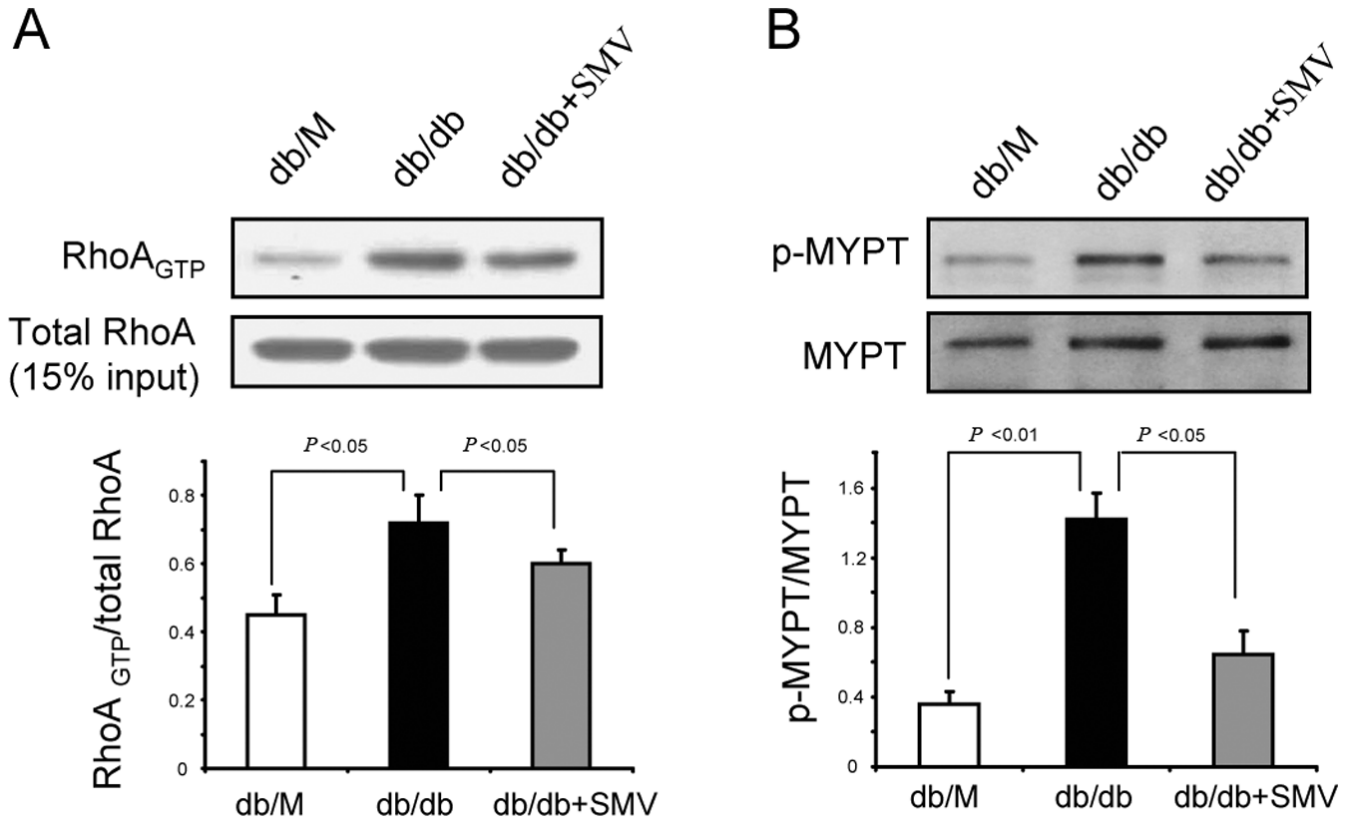
Simvastatin prevents ROCK1 activation in the glomeruli of mice with early-stage DN. A: Tissue lysate (300 µg) of glomeruli from mice (Figure 6A) were assessed for RhoA activity using the PEK-agarose pull-down assay. Fifteen percent of total protein lysate from each sample served as the loading control. Data are presented as means ± SEMs (*n* = 7). B: A total of 30 µg of glomerular lysate from each mouse was subjected to immunoblotting to assess ROCK1 activation. ROCK1 activity is presented as the p-MYPT1/MYPT1 ratio. Data are presented as means ± SEMs (*n* = 7).

Our *in vitro* studies indicated that ROCK1 mediates HG-induced dysregulation of occludin in GEnCs (Figure 4). Thus, we evaluated RhoA/ROCK1 activation in the glomeruli of db/db mice and the effect of simvastatin on ROCK1 activation in these mice as they developed early-stage DN. In isolated glomeruli of db/db mice, RhoA activity was greater than in control mice; moreover, simvastatin partially but significantly suppressed the activation of RhoA (Figure 7A). This suggests that RhoA activation underlies the activation of ROCK1. Then we assessed ROCK1 activation in isolated glomeruli of control and db/db mice. As expected, glomerular ROCK1 activity in db/db mice was higher than that in controls, consistent with a previous report [34]. Simvastatin treatment largely prevented the induction of ROCK1 in the glomeruli of db/db mice (Figure 7B). Notably, simvastatin led to a greater suppression of ROCK1 than RhoA, in agreement with our *in vitro* findings (Figure 4B). These results demonstrated that RhoA and additional mechanisms underlie the activation of glomerular ROCK during early-stage DN.

## Discussion

A large body of evidence suggests that many complications of diabetes, including DN, are related to cardiovascular disorders [35], [36]. Occludin and ZO-1 are the major components of endothelial TJ, and TJ dysfunction in endothelial cells contributes to hyperpermeability and other pathophysiological conditions [37]. In experimental models of diabetes, TJ dysfunction has been mainly examined in retinopathy or changes in the blood-brain barrier in the presence of diabetes mellitus [6], [38], [39]. Little is known about whether hyperglycemia causes TJ dysfunction in glomerular endothelial cells (GEnCs), especially before the development of mesangial expansion and glomerular sclerosis (*i.e.* early-stage DN). During early-stage DN, microalbuminuria usually indicates damage of the endothelium or podocytes without significant glomerular lesions. Prevention of the onset of microalbuminuria is usually considered the primary method for prevention of DN, because there is no loss of filtrate and patients respond well to prophylactic treatment at this early stage [2]. We used an established mouse model of early-stage DN [33] and demonstrated that occludin and ZO-1 were dysregulated in the GEnCs (Fig 5). We also provided evidence that activation of RhoA/ROCK1 signaling leads to TJ dysregulation in GEnCs. Furthermore, we found that the early abnormalities of GEnCs, as well as albuminuria, can be reversed by simvastatin treatment.

Previous studies of cultured endothelial cells indicated that high glucose induces degradation of TJ components and disruption of translocation [40], [41]. Although these findings clearly demonstrated that hyperglycemia leads to TJ dysfunction and hyperpermeability in cultured endothelial cells, the occurrence of these during early-stage DN has not been investigated. We demonstrated that hyperglycemia significantly repressed occludin expression, which is associated with dysfunctional translocation of occludin and ZO-1 in db/db mice with early-stage of DN, prior to mesangial expansion. Recognizing that occludin is expressed in podocytes and GEnCs, we considered the occludin signal observed in western blotting and immunostaining experiments as from both endothelial cells and podocytes (Figure 5). Based on the study of Fukasawa et al. [42], occludin expression in podocytes and endothelia is identical. However, in response to injury, the expression of podocyte occludin may differ from its expression in endothelial cells. According to Fukasawa et al., podocyte occludin is up-regulated to counteract the detachment induced by detrimental stimuli [43]. In contrast, we found that endothelial occludin expression is down-regulated in response to hyperglycemia. Although there is no data available on expression of podocyte occludin in response to hyperglycemia, its expression is presumably elevated. Thus, the decreased expression of glomerular occludin of mice with DN is mainly due to the down-regulation of endothelial occludin. Albuminuria was initially observed at 10 weeks in these mice, so this correlation implies that dysregulation of endothelial occludin/ZO-1 might be one of the mechanisms underlying microalbuminuria in early-stage DN.

Previous studies reported that statins may improve endothelial function [15], [44]. The current study extends these findings by demonstrating that simvastatin ameliorates glomerular endothelial dysfunction in early-stage of DN. This is relevant because clinical studies have shown that therapeutic interventions rarely improve kidney function in patients with advanced-stage DN [45]. In this study, we demonstrated that simvastatin significantly ameliorated the dysregulation of occludin/ZO-1 that is present at early-stage DN (Fig. 6) and efficiently prevented albuminuria in early-stage DN. This raises the possibility that statins could be used in clinical practice to prevent micro-albuminuria and the progression of early-stage DN.

Activation of RhoA is associated with cytoskeletal reorganization and the redistribution of occludin from the plasma membrane to the cytosol [37], [46]. Even though occludin binds directly to actin, this interaction is mediated by the ZO-1 protein [47]. Thus, it is possible that in addition to inhibiting mevalonate synthesis, statins also inhibit the synthesis of isoprenoid intermediates, and thereby prevent isoprenylation of RhoA and ROCK1 activation, thereby enabling occludin and ZO-1 to move to the plasma membrane.

Post-translational modifications of occludin, including proteolysis, phosphorylation, dimerization, and ubiquitination, are critical for maintaining its function and cellular concentration [48], [49]. For example, disruption of occludin translocation can induce degradation in an endocytosis-dependent manner [50], [51]. We found that the level of occludin protein was decreased by HG treatment, and this was accompanied by disruption of occludin and ZO-1 translocation (Figs. 1 and 5). Therefore, we speculate that deceased expression of occludin might be due to its increased degradation. ZO-1 regulates TJ function by directly binding to occludin or other TJ proteins (such as claudin-1) [52], [53]. If the ZO-1/occludin interaction does not form a stable TJ complex, it will trigger a rapid degradation of occludin but not ZO-1 [53]. This may explain why HG only decreased the protein level of occludin but not of ZO-1 (Fig. 1A). The assembly of the TJ also requires an interaction of ZO-1 with the actin cytoskeleton, and this is responsible for translocation of the occludin-ZO-1 complex to the plasma membrane [53]. The dynamics of the actin cytoskeleton is controlled by RhoA and other small GTPase signaling proteins [54]. As a downstream effector of RhoA, ROCK1 can interfere with the dynamics of the actin cytoskeleton by inhibition of myosin-light-chain (MLC) phosphatase [55]. We found that HG-stimulated down-regulation of occludin was concomitant with an increase in RhoA/ROCK1 signaling both *in vivo* and *in vitro* (Figs. 4 and 7). Use of siRNA to knockdown ROCK1, we further demonstrated that ROCK1 signaling is responsible for HG-induced dysregulation of occludin/ZO-1(Fig. 3). Thus, ROCK1 activation appears to be a critical event during early-stage DN. Similarity, simvastatin significantly preserved the level of p-MYPT1 in spite of hyperglycemia (Fig. 3). These findings indicate that RhoA/ROCK1 signaling regulates the function of the glomerular barrier, at least in part by interfering occludin/ZO-1 expression and translocation.

What factors could be responsible for the HG-induced activation of ROCK1 in GEnCs? It is well-known that Rho A small GTPase is an upstream activator of ROCK1 [55]. Indeed, we found that HG stimulates RhoA activity (Fig 4A). However, when we forced-expressed domain-negative RhoA in GEnCs, ROCK1 activity was only partially blunted (Fig 4.C). This finding raises the possibility that alternative pathways might be involved in the HG-induced activation of ROCK1. One possibility is that caspase-3 may be involved. It is known that caspase-3 can activate ROCK1 by removing the inhibitory Cy domain in ROCK1 [56] and that HG induces apoptosis and activation of caspase-3 in GEnCs [57]. Moreover, ROCK1 can activate PTEN, which in turn suppresses Akt activity and promotes caspase-3 activation [58]. Therefore, it seems possible that PTEN/Akt/caspase-3 might form a feed-back loop that further activates ROCK1 in GEnCs [59]. Many of the protective effects of statins are due to their ability to activate Akt [60], and activation of Akt should disrupt the PTEN/Akt/caspase-3 feed-back loop, leading to inhibition of ROCK1 activity (Figs. 4 and 7). This may explain why simvastatin can further suppress ROCK1 activity despite its inhibitory effect on RhoA.

In conclusion, we demonstrated that dysregulation of occludin and ZO-1 in GEnCs is an important pathological change present in early-stage DN. Activation RhoA/ROCK1 signaling by high glucose disrupts the translocation of occludin/ZO-1 and results in the loss of occludin. Simvastatin prevents the dysregulation of occludin/ZO-1 and the development of albuminuria by suppressing RhoA/ROCK1 signaling. These results suggest a potential therapeutic strategy for prevention of the onset of albuminuria in early-stage DN.
